# Apoptotic breast cancer cells after chemotherapy induce pro-tumour extracellular vesicles via LAP-competent macrophages

**DOI:** 10.1016/j.redox.2024.103485

**Published:** 2024-12-28

**Authors:** Qi Zhang, Xiaodi Liu, Qiuxia Wei, Shiyu Xiong, Wanrong Luo, Yingshi zhou, Jincheng Cao, Xiaolin Xu, Rongbin Liu, Xinyu Tang, Wenyue Zhang, Baoming Luo

**Affiliations:** a Department of Ultrasound, Sun Yat-Sen Memorial Hospital, Sun Yat-Sen University, Guangzhou, 510120, China; b Guangdong Provincial Key Laboratory of Malignant Tumour Epigenetics and Gene Regulation, Sun Yat-Sen Memorial Hospital, Sun Yat-Sen University, Guangzhou, 510120, China; c Department of Ultrasound, The First Affiliated Hospital with Nanjing Medical University, Nanjing, 210029, China; d Department of Ultrasound, Laboratory of Ultrasound Imaging and Drug, West China Hospital, Sichuan University, Chengdu, 610041, China; e Department of Breast Surgery, Department of General Surgery, The First Affiliated Hospital with Nanjing Medical University, Nanjing, 210029, China; f Jiangsu Key Lab of Cancer Biomarkers, Prevention and Treatment, Jiangsu Collaborative Innovation Center For Cancer Personalized Medicine, School of Public Health, Nanjing Medical University, Nanjing, 211166, China

**Keywords:** LC3-associated phagocytosis, S100A11, Breast cancer, Chemotherapy

## Abstract

Chemotherapy is important in the systemic therapy for breast cancer. However, after chemotherapy, the left living tumour cells are more progressive. There is an urgent need to study the underlying mechanism which is still unclear to further improve the therapeutic efficacy of chemotherapy in breast cancer. Here we find a pro-tumour effect of the apoptotic cells induced by the chemotherapy, which is mediated by a new subset of macrophages undergoing LC3-associated phagocytosis (LAP). By transferring exosomal S100A11 into the living tumour cells after chemotherapy, the macrophage exhibits a more pro-tumour phenotype than classic M2-type macrophages. Moreover, S100A11 binds to IFITM3, inducing Akt phosphorylation of living tumour cells after chemotherapy, which promotes tumour progression. Of note, Akt inhibitor can enhance the therapeutic effcicay of chemotherapy in breast cancer. This study provides a novel mechanistic link between tumour-associated macrophages and breast cancer, uncovering Akt as a potential therapeutic target to improve chemotherapy efficacy.

## 1 Background

Breast cancer poses a significant threat to women's health. Reducing recurrence rates and improving clinical outcomes are current focus of treatment [1]. Systemic treatment plays a crucial role in the treatment of breast cancer, particularly chemotherapy. Neoadjuvant chemotherapy (NACT) has significantly increased the survival period of patients, but tumours reduced in size by NACT may have a higher likelihood of local recurrence following breast-conserving therapy compared to tumours of the same size in women who have not undergone NACT [2]. Moreover, chemotherapy can promote lung metastasis of breast cancer [3]. Chemotherapy seems to promote the progression of the left living tumour cells. To further improve the therapeutic efficacy of chemotherapy in breast cancer, there is an urgent need to study the underlying mechanism which is still unclear.

Macrophages, a kind of antigen presenting cells, serve as the important phagocytes to establish the subsequent immune responses. In the tumour microenvironment, tumour-associated macrophages (TAMs) can be generally divided into M1-type (anti-tumour) or M2-type (pro-tumour). Recent studies have revealed that chemotherapy promotes the recruitment and infiltration of M2-type macrophages [4,5], thereby inducing vascular reconstruction, immunosuppression and ultimately tumour recurrence [6]. Our previous studies also reported that macrophages undergoing LC3-associated phagocytosis (LAP) tend towards an M2 polarized state [7], leading to pro-tumour immunosuppression [8]. LAP, an atypical form of phagocytosis that uses components of the autophagy pathway, has proven to be an effective defence against pathogens [9]. Autophagy is known to perform dual functions of substance engulfment and secretion mediation. Specifically, secretory autophagy serves as an alternative mechanism in extracellular secretion [10], to some extent aiding in the progression and metastasis of malignancy [11]. Extracellular vesicles (EV), including exosomes, as part of effective intercellular signalling systems, have also been identified as crucial agents for tumour metastasis [12].

S100 calcium-binding protein A11 (S100A11) is a member of the S100 protein family. A previous study demonstrated the dual role of S100A11 in tumour metastasis arrest and chemotherapeutic response improvement in gastric cancer [13]. Studies also noted that the dysregulation of S100A11/ANXA2, one node of a tumour suppressor/oncogene network, was found in the early stage of steatosis and was involved in the development of inflammation and liver cancer [14]. Moreover, S100A11 overexpression was found to promote pancreatic ductal adenocarcinoma (PDAC) progression by activating the pentose phosphate pathway [15]. However, the underlying mechanism of S100A11 in the progression of breast cancer has not yet been elucidated.

In this study, for the first time, we report that the dying tumour cells induce chemoresistance in the living tumour cells mediated by macrophages. A specific phenotype of macrophages has been found in breast cancer after chemotherapy, which occurs after engulfing dying tumour cells via LAP. Compared to M0 or M2 type macrophages, LAP-competent macrophages (LAP-M) exhibited an enhanced pro-tumour phenotype mediated by the release of exosomal S100A11. Moreover, exosomal S100A11 activated IFITM3/Akt passway in tumour cells. Of note, Akt inhibitor can enhance the therapeutic effcicay of chemotherapy in breast cancer. This study provides a novel mechanistic link between TAMs and breast cancer, uncovering Akt as a potential therapeutic target to improve chemotherapy efficacy.

## 2 Results

### 2.1 Increased infiltration of M2-like macrophages among chemotherapy resistance in breast cancer

**Fig. 1:**
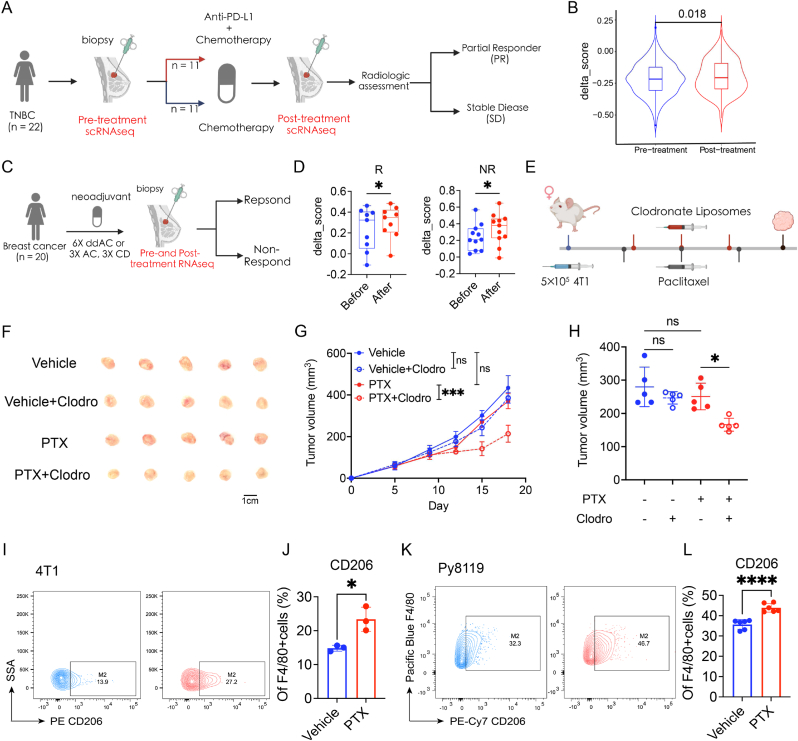
Increased infiltration of M2-like macrophages among chemotherapy resistance in breast cancer. **A** Schematic overview of the experimental design as the study reported. **B** Delta score (M2 score - M1 score) of macrophage in pre and post treatment of PTX. **C** Schematic overview of the experimental design as the study reported. **D** Bar plots showing delta scores in patients before and after chemotherapy grouped by response (n = 9 in R, n = 11 in NR). R, respond; NR, Non-respond. **E** Schematic diagram of administration of PTX and Clodro in 4T1-bearing mouse model. **F–H** Growth curve (G), tumour morphology (F) and bar plots of tumour weight (H) in four groups. **I-L** Representative flow plots of flow cytometry analysis and bar plots showing proprotions of CD206+macrophages in 4T1 and Py8119 tumours. Data are shown as mean ± SD (G-L), P > 0.05 (ns), P < 0.05 (∗), P < 0.01 (∗∗), P < 0.001 (∗∗∗), P < 0.0001 (∗∗∗∗).

To decode the changes in the transcriptional signature of macrophages after paclitaxel (PTX), single-cell RNA-seq data of breast tumour tissues before and after PTX were obtained (Fig. 1A) [16]. Delta score (M2 score - M1 score) was defined to investigate the differentiation bias of macrophages after PTX [17]. Patients with stable disease (SD) were selected because tumour volume is considered to influence the phenotype of macrophages in the tumour microenvironment. The results showed that, after PTX, the delta score increased, indicating a differentiation bias of M2 phenotype among patients with SD (Fig. 1B). To validate our results, we employed a transcriptomic dataset containing 20 breast cancer patients undergoing chemotherapy. Our analysis demonstrated that both chemotherapy responders and non-responders exhibited elevated delta scores after chemotherapy (Fig. 1C and D). Furthermore, in the Metabric dataset, Kaplan-Meier analysis demonstrated that higher delta scores were associated with worse overall survival in breast cancer patients undergoing chemotherapy (Fig. S1). Together, these data suggest that treatment with chemotherapy is associated with increased M2-like phenotype in breast cancer, which correlates to chemotherapy resistance.

To further elucidate the underlying relationship between macrophages and chemotherapy in breast cancer, BALB/c mouse-derived breast carcinoma cell line 4T1 and C57BL/6 mouse-derived breast carcinoma cell line Py8119 were utilized in our study and PTX was used for chemotherapy. We next evaluated immune infiltration in 4T1-bearing mice after chemotherapy using a mouse model as described in a previous study [3], in which the tumour volume showed no significant change. Clodronate liposomes (Clodro) were used for macrophage depletion (Fig. 1E). Based on the growth curve, tumour morphology, and tumour weight, we observed that Clodro significantly improved the therapeutic efficacy of PTX (Fig. 1F–H). Additionally, flow cytometric analysis also demonstrated that M2-like macrophages increased after chemotherapy in both the 4T1 and Py8119 breast cancer models (Fig. 1I–L). These findings collectively indicate that chemotherapy induces M2-like macrophages in breast cancer, without which chemotherapy exhibits better treatment outcomes.

### 2.2 Macrophages engulf chemotherapy-induced apoptotic cells through LAP in breast cancer

**Fig. 2:**
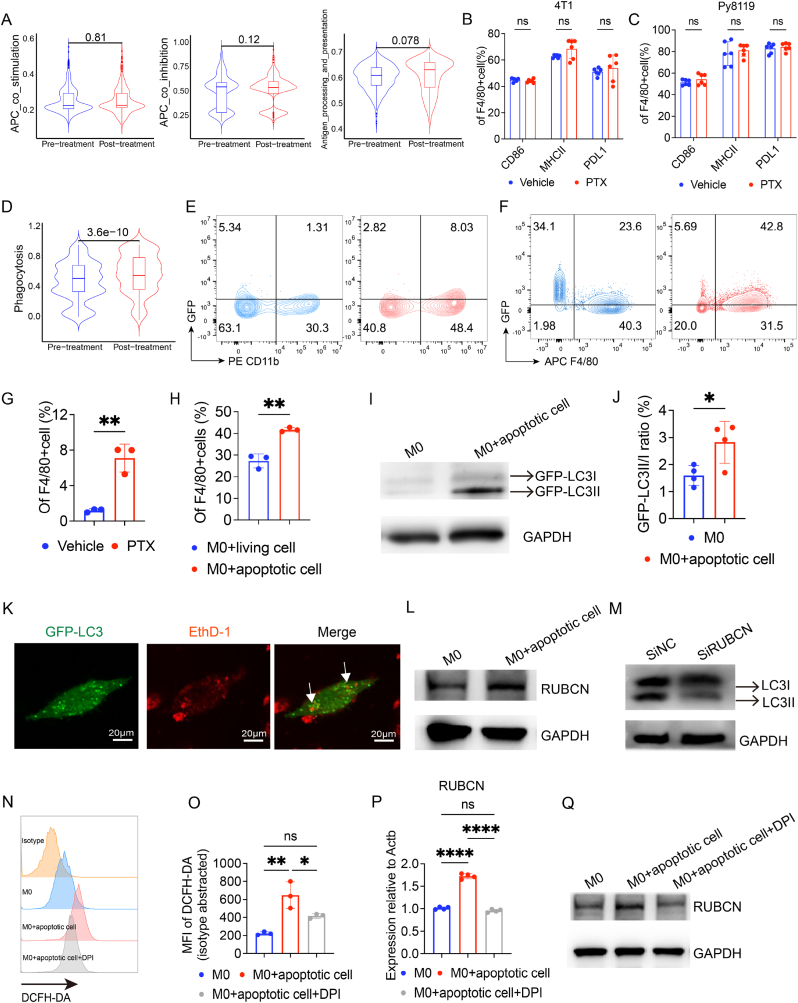
Macrophages engulf chemotherapy-induced apoptotic cells through LAP in breast cancer **A** Violin plots showing enrichment scores of macrophages before and after chemotherapy. **B–C** Bar plots showing proportions of macrophage subset in 4T1 and Py8119 models (n = 6). **D** Violin plots showing phagocytosing of macrophages before and after chemotherapy. **E-F** Representative flow cytometric plots illustrating macrophage phagocytosis of PTX‐treated GFP‐transfected 4T1 cells in vitro and in vivo (n = 3). **G-H** Bar plots of proportion in phagocytosed BMDMs (n = 3). **I-J** Lysates from BMDMs expressing GFP‐LC3 were immunoblotted with antibody to GFP (n = 3). Representative images of the Western blot were shown, and grey values were calculated. **K** Representative confocal images of Ethd‐1 labelled apoptotic 4T1 tumour cells (red) in BMDMs expressing GFP‐LC3 (green). **L** Lysates from BMDMs co-cultured with apoptotic cells or not were immunoblotted with antibodies to RUBCN. **M** Lysates from BMDMs transfected with siRUBCN were immunoblotted with antibodies to LC3B (n = 3). **N–O** Histogram and bar plots showed the mean fluorescence intensity (MFI) of DCFH-DA of BMDMs significantly increased after 30 min co-incubation with apoptotic cells, weakened by pre-treated with DPI (10 μM) for 1 h (n = 3). **P-Q** Bar plots showing mRNA expression of RUBCN (n = 3) and representative image of Western blot showing expression of RUBCN protein in BMDMs. Data are shown as mean ± SD (B-C and G-P), P > 0.05 (ns), P < 0.05 (∗), P < 0.01 (∗∗), P < 0.001 (∗∗∗), P < 0.0001 (∗∗∗∗).

Macrophages play a pivotal role in regulating CD8+T-cell-mediated anti-tumour responses. We next explored the alterations of CD8+T cells after chemotherapy from single-cell RNA-seq data. In contrast to the increased pro-tumour phenotype of macrophages after chemotherapy, CXCL13+CD8+T cells (tumour-reactive CD8+T cells) exhibited elevated cytotoxicity, inflammation, and activation scores after chemotherapy (Figs. S2A–C), which is in line with the elevated antigen presentation scores in dendritic cells after chemotherapy (Figs. S2D–F). These converse findings prompted us to comprehensively investigate the phenotype changes in macrophages induced by chemotherapy. Interestingly, although the enrichment scores of co-inhibition, co-stimulation, and antigen presentation in macrophages exhibited no significant changes (Fig. 2A), phagocytosis scores increased after chemotherapy (Fig. 2D), which is contradictory to the low capacity of phagocytosis in M2-type macrophages. These results were further validated by in-vivo experiments of 4T1 and Py8119 breast cancer models (Fig. 2B, C, E, and G). Specifically, no significant difference in CD86, MHCII, and PDL1 expression was observed between the vehicle and PTX groups (Fig. 2B and C). Additionally, GFP+4T1 tumour-bearing mice treated with PTX demonstrated an increase in macrophage phagocytosis of GFP+tumour cells (Fig. 2E and G). Our previous study demonstrated that macrophages, in a manner of LAP, phagocytosing dying cells were polarized towards M2-like phenotype (LAP competent macrophages, LAP-M) in incomplete radiofrequency ablation of hepatocellular carcinoma (HCC) [7]. In our study, chemotherapy could induce cell apoptosis, which was illustrated by Annexin V and PI staining and evaluated by Western blot with a cleaved caspase-3 antibody (Figs. S3A–C). Moreover, bone marrow-derived macrophages (BMDMs, Fig. S3D) exhibited an increase in phagocytic GFP+apoptotic cells compared to GFP+living cells (Fig. 2F and H). These findings collectively suggest that after chemotherapy, macrophages may engulf treatment-induced apoptotic tumour cells and subsequently differentiate into LAP-M.

Supporting this, we observed higher LC3II level (LAP-M marker) in BMDMs after co-culturing for 2 h with apoptotic cells (Fig. S3E), which is consistent with LAP-M reported in the literature [18]. To test whether the elevated LC3II in macrophages was attributed to additional uptake of LC3II from engulfed apoptotic cells (Fig. S3E), the GFP-LC3 knock-in reporter system in BMDMs was used, which allowed us to evaluate the de novo expressed LC3II in macrophages. By detecting GFP, A similar increase in GFP-LC3II was observed (Fig. 2I and J). Additionally, the laser scanning confocal microscopy (LSCM) assay showed that apoptotic cells colocalized with the LC3+compartment after 2 h co-culture, indicating the phagosomes formed [18] (Fig. 2K, arrowheads). It is well-known that LAP needs the participation of Rubicon (RUBCN), a key autophagy-associated gene, with reactive oxygen species (ROS) produced by NADPH oxidase 2 (Nox2) [19]. Consistently, co-culture with apoptotic tumour cells promoted the expression of RUBCN in macrophages compared with M0 group (Fig. 2L). When silencing RUBCN in BMDMs (Figs. S3F–H), LC3II protein level decreased obviously (Fig. 2M and S3I). In addition, ROS was generated in BMDMs after being stimulated with apoptotic cells (Fig. 2N and O) while diphenyleneiodonium chloride (DPI), a Nox2 inhibitor, downregulated the expression of RUBCN in BMDMs co-cultured with apoptotic cells (Fig. 2P, Q and S3J). The above results confirm the LAP in macrophages phagocytosing chemotherapy-induced apoptotic tumour cells, which is RUBCN and Nox2 dependent.

### 2.3 LAP-competent macrophages exerted a more pronounced pro-tumour effect than classic M2-macrophages in breast cancer

**Fig. 3:**
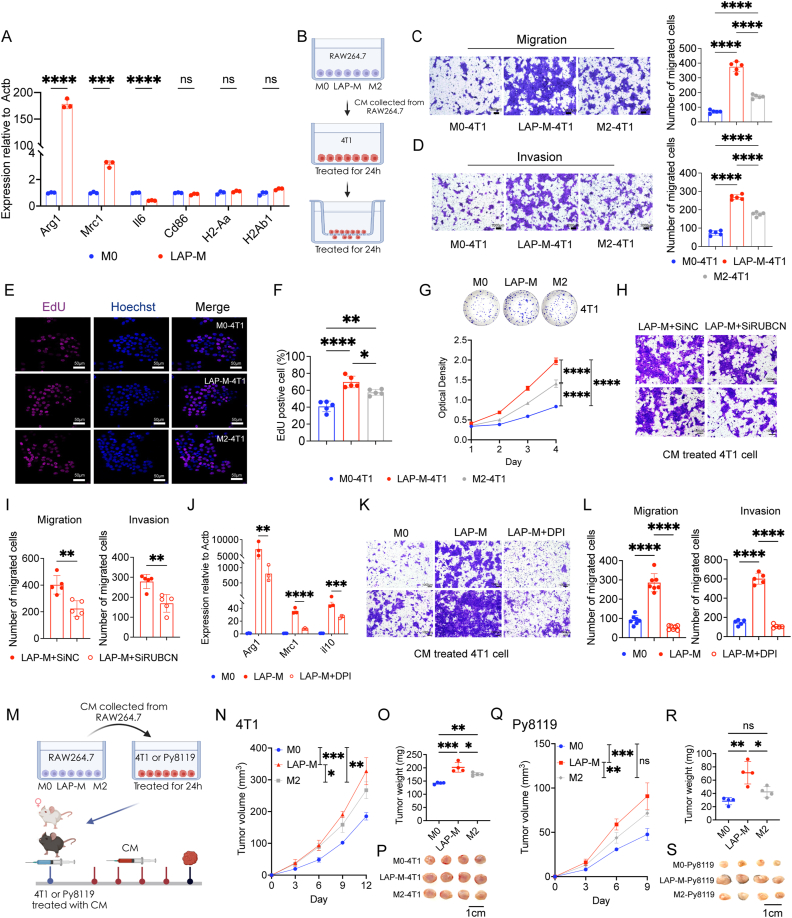
LAP-competent macrophages exerted a more pronounced pro-tumour effect than classic M2-macrophages in breast cancer **A** Bar plot showing gene expression in LAP-M and M0 macrophages by qPCR (n = 3). **B** Schematic diagram of co-cultured models in vitro. CM was collected after culturing different macrophages for 24 h for tumour cell culture. 24 h later, tumour cells were collected for further functional assays (C–I and K-L). **C-D** Representative images of tumour invasion and migration assays with the related bar plots shown in the right panel (n = 5). **E**-**F** Representative images of EdU assay with the related bar plots shown in the right panel (n = 5). **G** Representative images of plate cloning assay and line chart showing the optical density by CCK-8 assay (n = 6). **H–I** Counts of migrated tumour cells in migration (top) and invasion (bottom) assays of tumour cells treated with CM of macrophages after silencing RUBCN (n = 5). **J** Bar plot showing gene expression in LAP-M, M0, and LAP-M pre-treated with DPI for 1 h by qPCR (n = 3). **K-L** Counts of migrated tumour cells in migration (top) and invasion (bottom) assays of tumour cells treated with CM of LAP-M pretreated with DPI for 1h (n = 5). **M** Schematic diagram showing the experimental design of the in vivo experiments. Tumour cells were injected subcutaneously, and CM was injected intravenously every 2 days. **N–R** Tumour growth curves and weight in 4T1 (N–P) or Py8119 (Q–S) models (n = 4). Data are shown as mean ± SD, P > 0.05 (ns), P < 0.05 (∗), P < 0.01 (∗∗), P < 0.001 (∗∗∗), P < 0.0001 (∗∗∗∗).

To further determine whether M0 macrophages co-cultured with apoptotic cells exhibited M2 phenotype compared to M0, as observed in our in-vivo experiments, we evaluated the expression of various macrophage phenotype markers. As anticipated, the expression of M2 phenotype markers was upregulated (Arg1, Mrc1), but not markers linked to M1, antigen presentation, and co-stimulation phenotype (Fig. 3A) by qPCR. Similar results were observed with the LSCM and flow cytometry assays (Figs. S4A–C). These findings further validate the differentiation of LAP-M after engulfing apoptotic tumour cells. Previous studies have mainly explored the biological function of M2-type macrophages driven by IL‐4 or IL‐13, and macrophages undergoing LAP also exhibit a tumour-promoting phenotype [7,8]. RAW264.7 cell, a classic and recognized cell line for macrophage research was used for functional experiments. Interestingly, LAP-M showed a potent acceleration of 4T1 cell progression compared to the M2 phenotype polarized by IL-4, including invasion, migration (Fig. 3B–D, Figs. S4D–E), and proliferation (Fig. 3E–G), except for cell cycle analysis (Fig. S5). Additionally, siRUBCN and DPI significantly reduced the pro-tumour effect and M2-like phenotype of LAP-M (Fig. 3H–L and S6A-G). Notably, tumour cells treated with conditional medium (CM) from LAP-M exhibited increased tumour growth and elevated tumour weight compared to those stimulated with CM from M2-type macrophages (Fig. 3M–R). These findings suggest that LAP-M exhibits a more pronounced pro-tumour effect than M2-type macrophages.

### 2.4 LAP-competent macrophages promote 4T1 cell progression via EVs

**Fig. 4:**
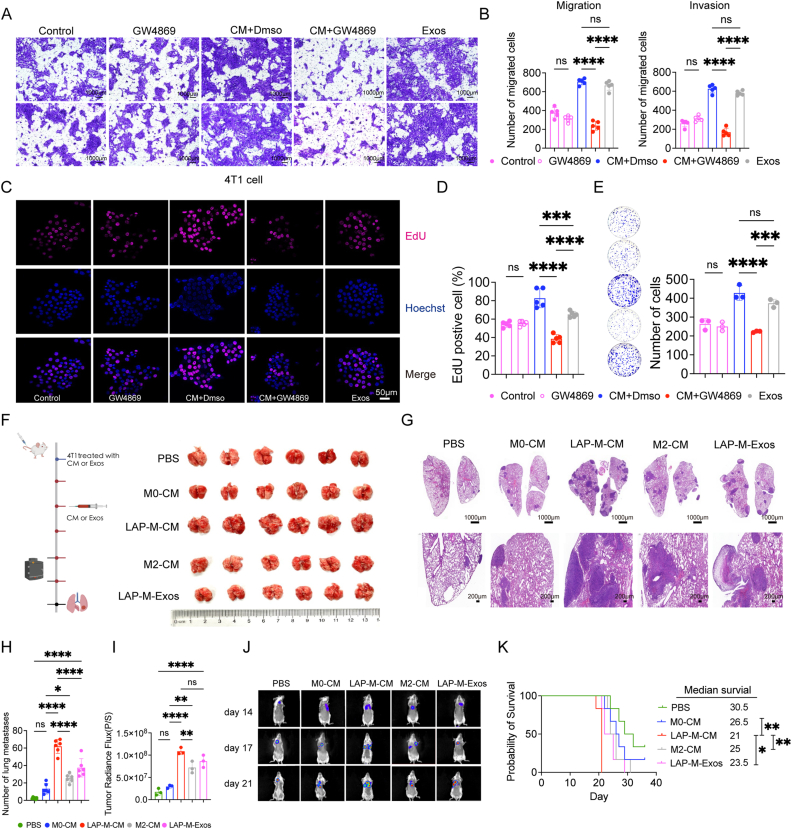
LAP-competent macrophages promote 4T1 cell progression via EVs. **A-B** Representative images of tumour invasion (bottom) and migration (top) assays (A) with the related bar plots (B), in which RAW264.7 cells was pretreated with GW4869 (10 μM for 24 h, n = 5). **C-D** Representative images of EdU assay (C) with the related bar plots (D) shown in the right panel (n = 5). **E** Representative images of colony formation assays with the related bar plots shown in the right panel (n = 3). **F** Schematic diagram of mice lung metastatic model. 4T1 cells were intravenously administered to mice after pretreatment with CM or exosomes twice a week. right**,** gross samples of lungs in five groups. **G** Representative HE sections of lungs in the five groups. Scale bars = 1000 μm and 200 μm. Exos, exosomes. **H** Bar plots of mouse lung metastases of five groups (n = 6). **I** Bar plots of luminescence intensity of five groups (n = 3). **J** Representative in vivo imaging system (IVIS) images detected at 14th, 17th and 21st days of BALB/c mice. **K** Survival analysis of five groups (n = 6). Data are shown as mean ± SD, P > 0.05 (ns), P < 0.05 (∗), P < 0.01 (∗∗), P < 0.001 (∗∗∗), P < 0.0001 (∗∗∗∗).

Secretory autophagy, a process that involves degradation, has been identified as an alternative mechanism for the extracellular secretion of molecules, including EVs [20,21]. Studies suggest that EVs play a pivotal role in intercellular communication, especially in tumour metastasis [22]. LC3-related secretion of proteins is secreted within small EVs (exosomes) but not other extracellular vesicles [20]. To investigate whether the robust tumour progression is associated with EVs, GW4869 [23], an inhibitor of exosome biogenesis and release, was utilized. We observed a noticeable reduction in 4T1 cell migration, invasion, and proliferation when adding GW4869 to the CM collected from M0 + apoptotic 4T1 cells. Importantly, GW4869 alone had no discernible impact on the migratory potential or proliferation ability of 4T1 cells. Direct supplementation of exosomes from LAP-M exhibited a similar pro-tumour effect to CM from LAP-M (Fig. 4A–E). Collectively, these findings suggest that exosomes are responsible for the pro-tumour effect of LAP-M on 4T1 cells.

To further investigate the pro-tumour function of the exosomes in vivo, a tumour model of lung metastasis which was widely used to test the in-vivo influence of exosomes on tumour cells was utilized in our study [11] (Fig. 4F). IVIS and HE analysis revealed more lung metastatic nodules in mice inoculated with 4T1 cells treated with LAP-M CM or exosomes (Fig. 4G–J). Similar trend was observed from survival analysis (Fig. 4K). The findings thus suggest that exosomes originating from LAP-M are key to the potent aggressiveness of 4T1 cells.

### 2.5 S100A11 is enriched in exosomes derived from LAP-competent macrophage in breast cancer

**Fig. 5:**
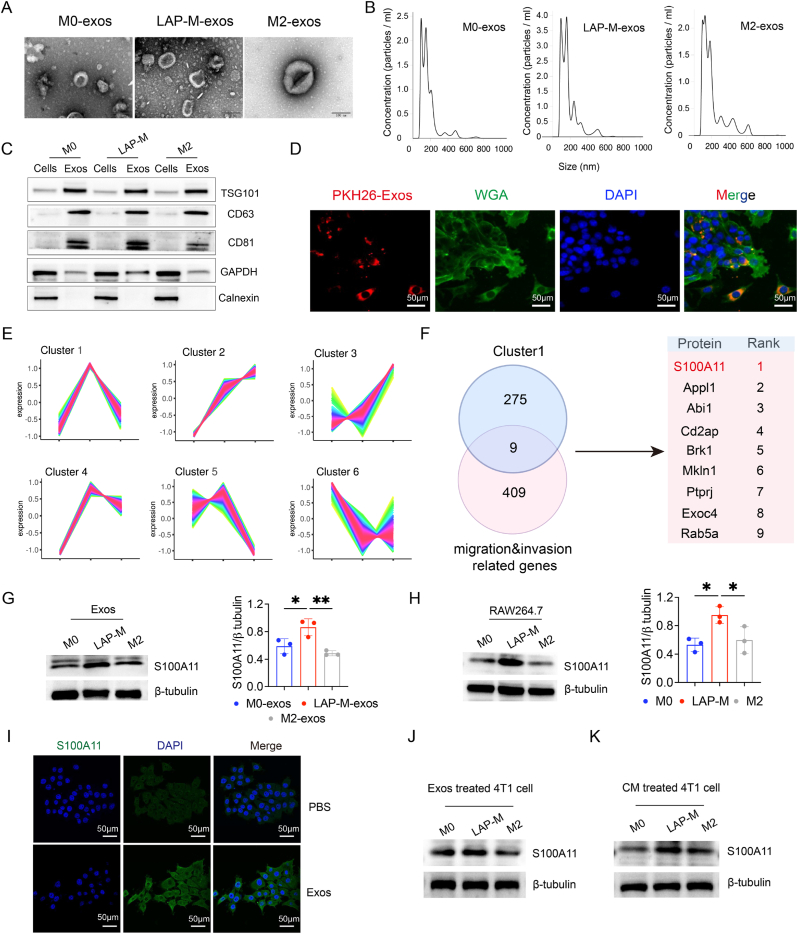
S100A11 is enriched in exosomes derived from LAP-competent macrophages in breast cancer. **A** Transmission electron microscopy images of vesicles originated from macrophages in three groups. **B** Nanoparticle tracking analysis of exosomes confirming the expected size range of 30–200 nm in diameter. **C** Lysates from exos and RAW264.7 cells were immunoblotted with antibodies to TSG101, CD63, CD81, GAPDH, and Calnexin. **D** Representative confocal images of uptake of PKH26-labelled exosomes (red) by 4T1 cell membrane stained with FITC-WGA (green), with DAPI staining used for the nucleus (blue). **E** Clustering analysis showed that proteins could be divided into 6 clusters with different trends (n = 3). **F** Venn plot showing that nine genes were identified in the intersection of two lists. **G-H** Lysates from exos (G) or RAW264.7 cells (H) were immunoblotted with antibody to S100A11 (left panel) and bar plots of grey value (right panel, n = 3). **I** Representative confocal images of exosomal S100A11 (green) absorbed in 4T1 cells after co-culture for 24 h. **J-K** Lysates of 4T1 cells treated with exosomes (J) or CM (K) from macrophages were immunoblotted with antibody to S100A11. Data are shown as mean ± SD, P > 0.05 (ns), P < 0.05 (∗), P < 0.01 (∗∗), P < 0.001 (∗∗∗), P < 0.0001 (∗∗∗∗).

Exosomes were successfully extracted from RAW264.7 cells. Vesicle morphology and particle size were validated by transmission electron microscopy (TEM) and Nanoparticles Tracking Analysis (Fig. 5A and B). Specifical exosome marker was also assessed by Western blot (Fig. 5C). LSCM assay showed that exosomes labelled by PKH26 (red fluorescence) could be absorbed by 4T1 cells, of which cell membrane was stained with Wheat Germ Agglutinin (WGA, green fluorescence), indicating exosomes could be transferred to 4T1 cells and then generated progressive potential. To identify specific molecules, we isolated and then analysed protein expression profiles of exosomes in three groups (Figs. S7A–B), including M0-exos (n = 3), LAP-M-exos (n = 3) and M2-exos (n = 3). The 4D label-free quantitative proteomics data identified six expression modules by unbiased clustering (Fig. 5E). We set the following screening criteria to meet our pro-tumour phenotype: ① the expression of exosome proteins in LAP-M-exos was much higher than that in M0-exos and M2-exos; ② M2-exos was slightly higher than M0-exos; ③ protein highly expressed in exosomes; ④ function as a pro-tumour factor. We thus focused on cluster 1, whose proteins met our criteria, involving a total of 284 different proteins. Considering our results that LAP-M markedly promoted migration and invasion of breast cancer cells, we employed a migration and invasion-related geneset with 418 genes from the MsigDB database (GO: 0031252). Venn analysis was performed between cluster 1 and this geneset, nine genes were identified in the intersection of the two data (Fig. 5F). Among them, S100A11 showed the highest expression level and was reported to regulate tumour cell invasion [24]. We subsequently verified the protein expression of S100A11 in exosomes of three groups by Western blot (Fig. 5G). A similar trend was observed in macrophage lysates (Fig. 5H). Furthermore, S100A11 carried in exosomes was verified to be transferred into 4T1 cells after co-culture (Fig. 5I–K). Together, these data suggest that S100A11 is highly expressed in LAP-M and transmitted intercellularly in the form of exosomes.

### 2.6 S100A11 in exosomes plays a key role in LAP-*M*-mediated 4T1 cell progression in breast cancer

**Fig. 6:**
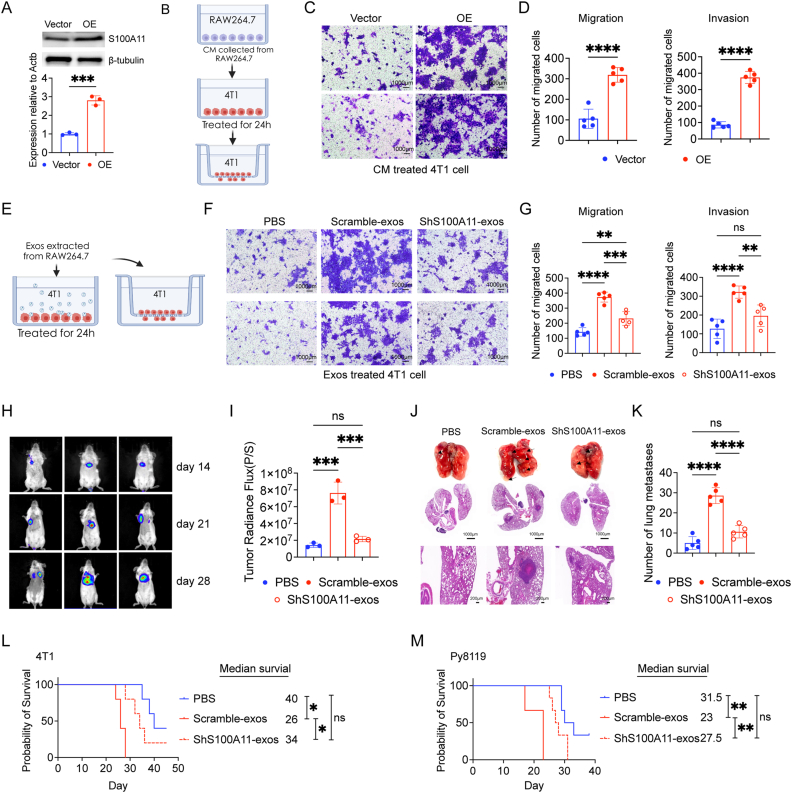
S100A11 in exosomes plays a key role in LAP-*M*-mediated 4T1 cell progression in breast cancer **A** Overexpression of S100A11 in RAW264.7 cells validated by qPCR (n = 3) and Western blot. **B** Schematic diagram of co-cultured models in vitro. CM was collected after culturing different macrophages for 24 h for tumour cell culture. 24 h later, tumour cells were collected for further functional assays (C and D). **C-D** Representative images of tumour invasion (bottom) and migration (top) assays after treatment with CM from macrophages overexpression of S100A11 (C), with the related bar plots (D, n = 5). **E** Schematic diagram of co-cultured models in vitro. Exos isolated from different macrophages were used to treat tumour cells. 24 h later, tumour cells were collected for further functional assays (F and G). **F-G** Representative images of tumour invasion (bottom) and migration (top) assays after treatment with exos from macrophages silenced by S100A11 (F), with the related bar plots shown in (G, n = 5). **H** Representative IVIS images detected at 14th, 21st and 28th days in BALB/c mice. Left, PBS; middle, Scramble-exos; right, ShS100A11-exos. **I** Statistical graph of luminescence intensity of metastatic nodules at 28th day (n = 3). **J** Lung tissue specimens and representative images of HE for lung metastatic nodules. **K** Bar plots of lung metastatic nodules (n = 5). **L-M** Survival analysis of mice with different treatments loaded with 4T1 (n = 5) or Py8119 tumours (n = 6). Data are shown as mean ± SD, P > 0.05 (ns), P < 0.05 (∗), P < 0.01 (∗∗), P < 0.001 (∗∗∗), P < 0.0001 (∗∗∗∗).

To investigate whether exosomal S100A11 is a potentially functional factor in progression, S100A11 was overexpressed (Fig. 6A) and knocked down in RAW264.7 cell (Figs. S8A–B) for further research. We subsequently repeated the functional assays in vitro. CM from RAW264.7 cells overexpressing S100A11 significantly enhanced the migration and invasion of 4T1 cells (Fig. 6B–D). Consistently, exosomes derived from macrophages with S100A11 knocked down could reduce the migration and invasion of 4T1 cell (Fig. 6E–G). Also, similar results were obtained in vivo with 4T1 model (Fig. 6H–K). Survival analysis showed that the mice survived longer after the knockdown of S100A11 (Fig. 6L and M). The above findings support that macrophage-derived exosomal S100A11 plays a key role in LAP-M mediated breast cancer progression.

### 2.7 Exosomal-S100A11 promotes progression of breast cancer by activating IFITM3/Akt pathway

**Fig. 7:**
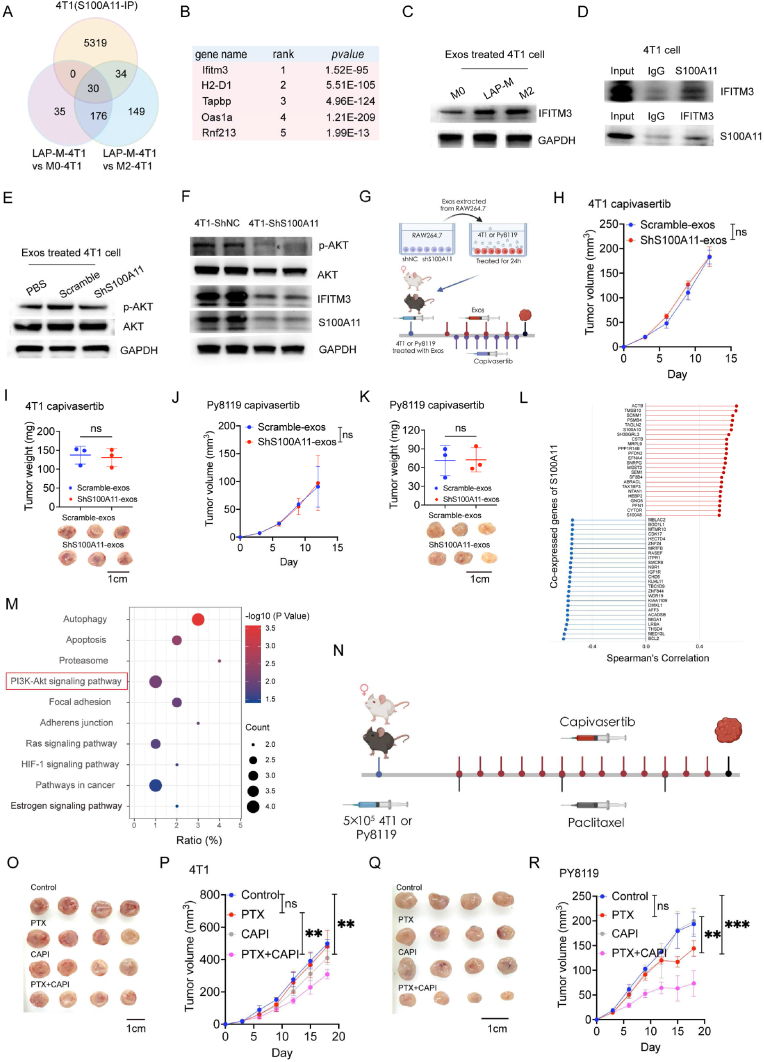
Exosomal-S100A11 promotes progression of breast cancer by activating IFITM3/Akt pathway **A** Venn diagrams of IP-MS (identifying the possible interacting proteins of S100A11) with differential expressed genes in RNA sequencing of LAP-*M*-4T1 vs M0-4T1 and LAP-*M*-4T1 vs M2-4T1. **B** The top five genes binding to S100A11 included IFITM3, which ranked first. **C** Lysates from 4T1 cells treated by exosomes from macrophages were immunoblotted with antibody to IFITM3. **D** Co-immunoprecipitation (Co-IP) had shown the binding of S100A11 and IFITM3 in 4T1 cells. **E** Lysates from 4T1 cells treated with exosomes from macrophages with S100A11 knocked down were immunoblotted with antibodies to *p*-AKT and AKT. **F** Lysates from 4T1 cells silenced with S100A11 were immunoblotted with antibodies to *p*-AKT, AKT, IFITM3, S100A11 and GAPDH. **G** Schematic diagram of in-vivo experiments. 4T1 or Py8119 cells were subcutaneously injected in mice after pretreatment with exosomes. Exosomes were intravenously injected twice a week and Capivasertib was administrated orally every day (H–K). **H–K** Tumour growth curves and weight of 4T1 (n = 3) or Py8119 models (n = 3). **L** The top 50 genes related to S100A11 in breast cancer from TCGA database. **M** Bubble map showed functional pathways related to S100A11 and PI3K-Akt signal pathway was marked by red. **N** Schematic diagram of in-vivo experiments. 4T1 or Py8119 cells were subcutaneously injected in mice. PTX was intraperitoneally injected every five days and Capivasertib was administrated orally every day (O–R). **O–R** Tumour growth curves of 4T1 (n = 4) or Py8119 models (n = 4). Data are shown as mean ± SD, P > 0.05 (ns), P < 0.05 (∗), P < 0.01 (∗∗), P < 0.001 (∗∗∗), P < 0.0001 (∗∗∗∗).

To further investigate the underlying mechanisms of the pro-tumour effect of exosomal S100A11, RNA sequencing was performed on the 4T1 cells co-cultured with M0 (M0-4T1), M2 (M2-4T1) or LAP-competent macrophages (LAP-*M*-4T1) and protein mass spectrometry (MS) was performed to detect proteins bound to S100A11 in 4T1 cells via immunoprecipitation (IP). Differentially expressed genes between LAP-4T1 and M0-4T1 or LAP-4T1 and M2-4T1 were used for Venn analysis (Fig. 7A). 30 genes were identified in the intersection of the three data (Fig. 7A). Ifitm3 was significantly upregulated, which might be potent downstream (Fig. 7B). The expression of IFITM3 was upregulated in 4T1 cells co-cultured with LAP-M (Fig. 7C). Moreover, IP experiments showed that IFITM3 bound to S100A11 in the 4T1 cells (Fig. 7D). Overall, the results indicated that S100A11 could regulate IFITM3 intracellularly.

Studies indicated that S100A11 could activate the PI3K-Akt pathway exhibiting significant biological activity [25]. Moreover, Akt activation was dependent on IFITM3 [26]. Thus, an investigation was performed to determine the alteration of Akt phosphorylation in 4T1 cells treated with ShS100A11-exos compared to those treated with Scramble-exos. Exosomes derived from ShS100A11 macrophages reduced the expression of *p*-AKT in 4T1 cells (Fig. 7E). To further validate our results, S100A11 was knocked down in 4T1 cells to directly reduce the intracellular S100A11. The results showed that lower intracellular S100A11 reduced IFITM3 expression and *p*-AKT in 4T1 cells (Fig. 7F). To further validate the pivotal role of Akt in LAP-M exosome-mediated breast cancer progression, exosome-treated tumour cells were inoculated subcutaneously in mice administrated with Akt inhibitor orally. Inhibition of Akt reduced the pro-tumour effect of exosomal S100A11 from LAP-M in both 4T1 and Py8119 models (Fig. 7H–K). Moreover, we utilized the TCGA database to analyze S100A11-coexpressed genes in breast cancer (Fig. 7L). Consistent with our results described above, the PI3K-Akt pathway was significantly enriched in these genes, as depicted in bubble plots (Fig. 7M). The other two enriched pathways, including Ras and HIF-1 pathways, are also related to Akt signaling [27,28]. The above findings illustrated that S100A11 regulated the IFITM3/Akt pathway intracellularly.

Capivasertib, a pan-Akt inhibitor, has been approved to treat hormone receptor-positive, human epidermal growth factor receptor 2-negative, locally advanced or metastatic breast cancer with PIK3CA/AKT1/PTEN alterations with Fulvestrant [29]. Notably, pharmacological Inhibition of Akt with Capivasertib significantly enhanced the therapeutic efficacy of PTX. Specifically, PTX did not impair tumour growth, while tumours treated with the combinational therapy of Capivasertib and PTX exhibited sigficantly reduced volume (Fig. 7N-R). This finding represents the combinational therapy of Capivasertib and chemotherapy as a potential therapeutic strategy for breast cancer.

### 2.8 S100A11 could be an underlying marker for predicting chemotherapy resistance in breast cancer

We next aimed to evaluate whether exosomal S100A11 could be a predictor of chemotherapy resistance in breast cancer. S100A11 expression was assessed by ELISA on extracted exosomes from peripheral blood. The results showed that more exosomal S100A11 was expressed in the plasma of 4T1-bearing mice after chemotherapy (Fig. S8). The results indicated that S100A11 might be an underlying marker for chemotherapy resistance prediction, although clinical validation is still needed.

## 3 Discussion

NACT has significantly increased the survival period of patients, but tumours reduced in size by NACT may have a higher likelihood of local recurrence following breast-conserving therapy compared to tumours of the same size in women who have not undergone NACT [2]. Studies have noted that chemotherapy can increase the recruitment and infiltration of TAMs in tumours, which is associated with a poor prognosis [30]. Similarly, we observed the aforementioned phenomenon, but the underlying mechanism needs further investigation. Classic M2-type macrophages are important pro-tumour composition in the tumour environment after chemotherapy [4]. This study first revealed that macrophages, which engulf tumour cells killed by chemotherapy via LAP exhibited a stronger pro-tumour effect than M2-type macrophages in vivo and in vitro. Previous studies have focused on the mechanisms of chemotherapy primary resistance. Instead, this study innovatively proposes a mechanism of pro-tumour effect after chemotherapy. According to our findings, LAP-M could be the therapeutic target but not M2, which is usually focused on in the clinical practice [31], to improve the efficacy of chemotherapy in breast cancer patients.

To investigate the internal mechanism, we directed our focus towards the secretion of EVs. LAP is not only an unconventional autophagy process but is also traditionally considered a lysosomal degradation process [32], which is associated with the production and release of EVs [33], referred to as secretory autophagy. Numerous reports support that exosomes released by either tumour or stromal cells have the ability to enhance the drug resistance of tumour cells by transferring nucleic acids, proteins and metabolites or expressing immunotherapy targets [34,35]. Guo J et al. noted that M2-type macrophages secreted exosomes enriched with miR-2-186p to promote colon cancer metastasis by regulating DLC1 [36]. Similarly, exosomes isolated from M2-type macrophages caused an imbalance in the TGF-β7/BMP-2 pathway and promoted the invasion of HCC cells [37].

To identify the key functional factors in exosomes derived from macrophages undergoing LAP, we performed proteomic sequencing analysis on exosomes and found that the S100 calcium-binding protein family was significantly upregulated in exosomes of macrophages undergoing LAP. Recent studies have revealed the pivotal involvement of the S100A family of proteins in cancer metastasis and premetastatic niche formation [38]. Ji X et al. identified S100A11 as a potential metastatic and prognostic factor in tumours [38]. Our research illustrated the critical function of exosomal S100A11 in governing the progression of breast cancer. S100A11 has the potential as a significant prognostic biomarker for chemotherapy-resistant patients as a higher expression in peripheral blood from tumour-bearing mice after chemotherapy. Exosomal S100A11 released by LAP-M contributed to progression of breast cancer by means of Akt phosphorylation, indicating of Akt as a promising target to reverse the pro-tumour effect induced by the dying tumour cells. Capivasertib, a pan-AKT inhibitor, has been approved to treat hormone receptor-positive, human epidermal growth factor receptor 2-negative, locally advanced or metastatic breast cancer with PIK3CA/AKT1/PTEN alterations with Fulvestrant [29]. Moreover, capivasertib significantly enhanced the efficacy of endocrine therapy for endocrine therapy-resistant breast cancer [39]. Our data revealed that Capivasertib significantly enhanced the therapeutic efficacy of chemotherapy in two breast cancer models. Thus, the combination of chemotherapy and capivasertib might be a promising strategy to further improve the prognosis of breast cancer patients, which needs clinical trials in the future.

In conclusion, we discovered that macrophages exhibited a LAP-competent phenotype after chemotherapy. Strategic targeting of macrophages can effectively inhibit the pro-tumour effects of TAMs while simultaneously promoting the cross-presentation of CD8+T cells and amplifying their anti-tumour effects [40], indicating that macrophages have potential as prognostic biomarkers and therapeutic targets. We demonstrated that macrophages underwent LAP to engulf apoptotic cells, released exosomes enriched with S100A11 and absorbed by breast cancer cells. And then S100A11 bound to IFITM3 and exerted the pro-tumour effect by activating Akt pathway. Of note, Akt inhibitor can enhance the therapeutic effcicay of chemotherapy in breast cancer. This study provides a novel mechanistic link between TAMs and breast cancer, representing Akt as a potential therapeutic target to improve chemotherapy efficacy.

Indeed, there are some limitations in this study: Firstly, we identified a mechanism by which LAP-M exerts its pro-tumour effect after chemotherapy in breast cancer. However, other factors detected in EVs may also play a pro-tumour effect in LAP-*M*-mediated tumour progression, which needs future investigation. Secondly, whether other chemical agents can induce the pro-tumour effect should be determined in the future. Thirdly, whether other vesicles secreted by LAP-M shows pro-tumour effect needs future exploration. Fourthly, because of lack of clinical samples, more clinical validation is in need in the future.

## 4 Methods

### 4.1 Antibodies and agents

The antibodies and agents utilized in this study were as follows: anti-LC3B (CST, Cat. No. 43566), anti-Rubicon (Proteintech, Cat. No. 21444-1-AP), anti-AKT (CST, Cat. No. 4691), anti-*p*AKT (CST, Cat. No. 4060), anti-GAPDH (Immunoway, Cat. No. YN5585), anti-β-Tubulin (Proteintech, Cat. No. 10068-1-AP), anti-S100A11 (Proteintech, Cat. No. 10237-1-AP), anti-IFITM3 (Proteintech, Cat. No. 11714-1-AP), Fixable Viability Dye eFluor™ 780 (eBioscience, Cat. No. 65-0865-14), PE anti-mouse/human CD11b (Biolegend, Cat. No. 101207), APC anti-mouse F4/80 (BD Bioscience, Cat. No. 566787), FITC anti-mouse F4/80 (Biolegend, Cat. No. 123107), APC anti-mouse CD206 (Biolegend, Cat. No. 141707), APC-Cy^TM^7 anti-mouse CD45 (BD Bioscience, Cat. No. 557659), BV421 anti-mouse CD206 (Biolegend, Cat. No. 141717), Pacific Blue™ anti-mouse F4/80 (Biolegend, Cat. No. 123123), PE anti-mouse CD206 (MMR) (Biolegend, Cat. No. 141705), Zombie Aqua™ Fixable Viability Kit (Biolegend, Cat. No. 423101), FITC anti-mouse CD45 (Biolegend, Cat. No. 157213), APC-cy7 anti-mouse CD86 (Biolegend, Cat. No. 105029), PE/Cyanine7 anti-mouse CD206 (Biolegend, Cat. No. 141719), PerCP/Cyanine5.5 anti-mouse I-A/I-E (Biolegend, Cat. No. 107625), PE anti-mouse PDL1 (Biolegend, Cat. No. 105029), Recombinant Mouse M-CSF Protein (Sino Biological, Cat. No. 51112-MNAH), Recombinant Mouse IL-4 (PeproTech, Cat. No. 214-14), GW4869 (Sigma, Cat. No. 6823-69-4), diphenyleneiodonium chloride (DPI) (Selleck, Cat. No. 4673-26-1), paclitaxel (PTX) (Selleck, Cat. No. 33069-62-4).

### 4.2 Cell lines

4T1, RAW264.7 and Py8119 cell lines were procured from the American Type Culture Collection (ATCC, USA). Bone marrow-derived macrophages (BMDMs) were extracted and cultivated using M-CSF (20 ng/mL) for 7 days to conduct further investigation. 4T1 cell lines comprising GFP were created using retroviral plasmid transduction. Moreover, macrophages that featured GFP-LC3 were transfected with retrovirus plasmids that expressed GFP-LC3 protein obtained from GeneChem, China.

### 4.3 Paclitaxel-treated 4T1 cell preparation

A total of 3 × 10^3^ cells were seeded into 96-well plates to conduct CCK-8 assays, while 2 × 10^5^ cells were seeded into 6-well plates (for the PI/Annexin V kit) overnight. Various concentrations (1.25, 2.5, 5, 7.5, 10 μg/mL) of PTX were added simultaneously and assessed after 12, 24 and 48 h. The concentration of dimethyl sulfoxide (DMSO) remained below 0.5 %. The cells were characterized by western blotting of cleaved-caspase-3 at a final concentration of 10 μg/mL.

### 4.4 Establishment of in vivo tumour models

To establish an orthotopic model, 5 × 10^5^ cells were inoculated under the fat pad of the fourth pair of mammary glands on the left side of 6- to 8-week-old female BALB/c mice. Following the successful establishment of the mouse model, the tumour size was measured utilizing vernier calipers every three days. Tumour volume was calculated by formula V=W^2^ × L/2 (where "W" refers to the tumour's short diameter and "L" to the tumour's long diameter). When tumour volume reached 50 mm^3^, the mice were randomly divided into groups. For PTX treatment, 10 mg/kg PTX was injected intraperitoneally every five days. For clodronate liposomes treatment, clodronate liposomes was injected intravenously every four days (200 μL for the first time and 150 μL for the next injection). For Capivasertib treatment, 100 mg/kg Capivasertib was orally administrated every day. For CM injection, 30 μL CM was injected intratumourly every two days.

To generate a mouse lung metastasis model, we first co-cultured 4T1 cells with either 10 μg/mL exosomes or CM for 24 h. Subsequently, 2 × 10^5^ 4T1 cells were injected intravenously, together with either 10 μg exosomes or 100 μL CM or PBS (as a control). Intravenous injection was conducted twice a week.

### 4.5 Isolation and identification of exosomes

RAW264.7 cells were cultured using DMEM supplemented with 10% FBS until they reached 80% confluence. Subsequently, the CM was removed, and the cells were washed with PBS before being incubated with exosome-depleted medium for 48 h. According to the published protocol [41], the supernatants were subjected to multiple centrifugation steps at varying speeds (300×*g* for 10 min, 2000×*g* for 10 min, and 10000×*g* for 30 min) at 4 °C to eliminate cells and cell debris. The resulting supernatants were then transferred to new ultracentrifuge tubes and subjected to a final centrifugation at 120000×*g* for 70 min at 4 °C. The resulting pellets were resuspended in 35 mL PBS, and exosomes were obtained by centrifugation at 120000×*g* for 70 min followed by resuspension in cold PBS.

### 4.6 Exosomes uptake assay

PKH-26 kit (Sigma, USA) was utilized to label exosomes in accordance with the manufacturer's protocol. The labelled exosomes were incubated with 4T1 cells within a confocal well plate for 8 h. After fixation with 4% paraformaldehyde, the cells were stained with WGA and DAPI. The image was analysed using LSCM.

### 4.7 Nanoparticle tracking analysis

Particle size and concentration were evaluated using a NanosightNS300 (Malvern, UK). Exosomes were appropriately diluted in PBS at a ratio of 1:1000, and the particle size distribution was measured at 25 °C.

### 4.8 Transmission electron microscopy

Exosome suspension solution was added onto the copper net and precipitated for 3 min, and the floating liquid was removed by filter paper. Phosphotungstic acid (0.2%) was subsequently loaded on the copper net, and the sample was precipitated for 3 min, dried at room temperature for 5 min, and then imaged by TEM (JEM-1200EX, JEOL, Japan).

### 4.9 4D label-free quantitative proteomics

Exosome proteins were extracted with SDT buffer (4% SDS, 100 mM Tris-HCl, pH 7.6), quantified with the BCA Protein Assay Kit (P0012, Beyotime) and then separated on a 12.5% SDS‒PAGE gel. After filter-aided sample preparation (FASP) digestion, eligible samples were analysed on a nanoElute (Bruker, Bremen, Germany) coupled to a timsTOF Pro (Bruker, Bremen, Germany). The MS data were processed using MaxQuant software (http://www.maxquant.org.) version 1.6.14.0. and then searched against the Mus musculus protein database in UniProt (January 2022). Protein abundance was calculated on the basis of the normalized spectral protein intensity (LFQ intensity) [42]. Proteins with a fold change (FC) > 1.5 and P value < 0.05 were screened and defined as differentially expressed proteins. Bioinformatics analyses were carried out with R version 3.3.1 (https://www.r-project.org/) statistical computing software.

### 4.10 Bulk RNA-seq analysis

Samples were subjected to RNA extraction using Trizol (Thermo Fisher Scientific, 15596018). Library preparation was performed using Bioanalyzer 2100 and RNA 6000 Nano LabChip Kit (Agilent, CA, USA, 5067-1511), high-quality RNA samples with RIN number > 7.0 were used to construct sequencing library. And then performed the 2 × 150bp paired-end sequencing (PE150) on an Illumina Novaseq™6000 following the vendor's recommended protocol. Genes differential expression analysis was performed by DESeq2 software between two different groups (and by edgeR between two samples). The selection criteria for significantly differentially expressed genes were: |log2FC| > 1.5 and P-value < 0.05.

Data published previously was used in this study [43]. Processed data were obtained from GSE191127 (GEO database). The enrichment score was calculated by R package GSVA (v 1.40.1).

### 4.11 Single-cell RNA seq analysis

Data published previously was used in this study [16]. As previously reported, we processed the raw data with quality control and performed dimensionality reduction, clustering, cell-type labeling, and visualization. The enrichment score was calculated by R package GSVA (v 1.40.1).

### 4.12 Survial analysis

Processed data of Metabric cohort were downloaded from http://www.cbioportal.org/. The macrophage polarization score was calculated by R package GSVA (v 1.40.1). Kaplan-Meier analysis was performed by R package (v).

### 4.13 Flow cytometry

Single-cell suspensions (10^5^ cells/tube) were obtained and subsequently resuspended in PBS. Cells were stained with FVD, FcR blocker firstly and then stained with CD45, CD11b, F4/80, CD206, CD86, I-A/I-E and PDL1 at 4 °C for 30 min, washed with PBS and resuspended in 500 μL staining buffer. Cellular subsets were sorted through FACSVerse (BD Biosciences, USA) and CytoFLEX (Beckman, USA). Data were analysed utilizing FlowJo Version 10.2 (FlowJo LLC, USA).

### 4.14 Immunoprecipitation and LC-MS/MS analysis

Immunoprecipitation was performed to isolate the target proteins from cell lysates. Briefly, cell lysates were incubated with S100A11 antibodies against the protein of interest, followed by the addition of protein A/G agarose beads to capture the antibody-protein complexes. After extensive washing to remove non-specific binding, the complexes were eluted and analysed by mass spectrometry to identify the target protein or its interacting partners.

Proteins (10 μg) were reduced with 5 mM dithiothreitol (DTT) and alkylated with 10 mM iodoacetamide. After dilution with 25 mM ammonium bicarbonate (ABC), trypsin (1:50) was added, and digestion was carried out overnight at 37 °C. The digestion was terminated with 50 μL 0.1% formic acid (FA), followed by desalting using a C18 column. The eluates were lyophilized and stored at −80 °C. Tryptic peptides (1 μg) were analysed by LC-MS/MS on an Orbitrap Eclipse mass spectrometer coupled with an EASY nLC 1200 system. Peptides were separated using a gradient from 8% to 40% solvent B (80% ACN, 0.1% FA) in 90 min. Data were processed using Proteome Discoverer, and protein identification and quantification were performed with Sequest HT against the UniProtKB Mus_musculus database, applying a false discovery rate (FDR) of 1%.

### 4.15 Western blotting

Total cell protein was extracted with RIPA lysis buffer (Beyotime, China), and the protein concentration was determined by bicinchoninic acid (BCA) assay (CWBIO, China). After separation by 4–20% SDS‒PAGE, the samples were transferred onto PVDF membranes (Millipore, USA). Antibody-bound protein bands were detected by chemiluminescence (BLT GelView 6000Pro, China). The greyscale value of the images was analysed by ImageJ software.

### 4.16 Quantitative real-time PCR (qRT‒PCR)

The RNA pure Tissue & Cell Kit (ESscience) was employed to isolate total RNA from cells. Isolated RNA was employed as a template for reverse transcription reactions with the Color Reverse Transcription Kit (EZBioscience). The quantitative amplification procedure was performed using 2 × SYBR Green qPCR Master mix (EZBioscience) in a LightCycler® 96 System. The primers used were sourced from Hongxun Biotechnologies, China.

### 4.17 Immunofluorescence

Immunofluorescence (IF) was utilized to stain cells with the appropriate antibodies, including anti-mouse S100A11and anti-mouse CD206 and F4/80.

### 4.18 Migration and invasion

Migration and invasion abilities were assessed by the transwell assay (Corning, USA). In brief, 600 μL of medium supplemented with 10% FBS was added to the lower chamber, while 200 μL of serum-free DMEM was added to the upper chamber. For invasion assays, a 1:10 dilution ratio of Matrigel (BD Bioscience, USA) was added to the upper chamber. Co-cultured cells were then seeded into the upper chamber at a concentration of 2.5 × 10^5^ cells/mL. Following incubation for 24 and 48 h at 37 °C, the bottom chamber was fixed using 4% polyoxymethylene and stained with 0.5% crystal violet (Servicebio, China). The stained cells were visualized using a microscope (Nikon, Japan).

### 4.19 Cell proliferation assay

A total of 1 × 10^3^ cells were seeded onto 96-well plates, with 5 replicates per condition, and cell proliferation was monitored using CCK-8 assay. Absorbance readings were taken at 24, 48, 72, and 96 h.

### 4.20 Colony formation assay

A total of 5 × 10^2^ cells were added to each well of a 6-well plate and grown for 7 days. Then, cell colonies were fixed with 4% paraformaldehyde, stained with crystal violet and imaged to analyze the number using vSpot Reader Spectrum (AID, Germany).

### 4.21 EdU proliferation assay

The BeyoClick™ EdU-488 kit (Beyotime, China) was used in accordance with the manufacturer's instructions to evaluate the proliferation activity of 4T1 cells after 24 h of culture with macrophage supernatants.

### 4.22 Plasmid and shRNA constructs and lentivirus packaging

The murine S100A11 overexpression plasmid was designed and constructed by Genepharma (Suzhou, China). Control and target-specific shRNA sequences were subsequently designed and obtained from OBiO Technology (Shanghai, China) [Scramble (5′-CCTAAGGTTAAGTCGCCCTCG-3′), shRNA-1 (5′-GCTAGATTTCCAAGAGTTTCT-3′), shRNA-2 (5′-GCTATAGCGTGCCATGATTCT-3′), and shRNA-3 (5′-GAAGGATCCTGGTGTCCTTGA-3′)]. Both qPCR and Western blotting were utilized to evaluate the efficiency of transfection and knockdown.

### 4.23 Statistical analysis and reproducibility

All comparisons were evaluated using Student's *t*-test or one-way ANOVA. Statistical analyses were performed using GraphPad Prism 9 and Rstudio, with significance determined as a p value < 0.05. All experiments were repeated for at least two times independently.

## CRediT authorship contribution statement

**Qi Zhang:** Investigation, Methodology, Writing – original draft. **Xiaodi Liu:** Conceptualization, Funding acquisition. **Qiuxia Wei:** Methodology. **Shiyu Xiong:** Investigation. **Wanrong Luo:** Methodology. **Yingshi zhou:** Investigation. **Jincheng Cao:** Investigation. **Xiaolin Xu:** Funding acquisition. **Rongbin Liu:** Funding acquisition. **Xinyu Tang:** Writing – review & editing. **Wenyue Zhang:** Conceptualization. **Baoming Luo:** Conceptualization, Supervision.

## Data availability statement

All data are available in the published article.

## Ethics approval statement

Animal experiments were performed under guidelines approved by the Ethics Committee for the Care and Use of Laboratory Animals of the Sun Yat‐Sen University (Approval number: SYSU-IACUC-2022-000920).

## Consent for publication

All authors have read and approved the publication of the manuscript.

## Funding statement

This work was supported by the 10.13039/501100001809National Natural Science Foundation of China (82171944, 82202162, 82202907) and 10.13039/501100003453Natural Science Foundation of Guangdong Province (2021A1515012611, 2023A1515012705) and 10.13039/501100002858China Postdoctoral Science Foundation (2023M732449) and Young Scholars Fostering Fund of the First Affiliated Hospital of 10.13039/501100007289Nanjing Medical University. The Figures of contents are created with BioRender.com. We thank staff at LC-Bio Technology for sequencing services.

## Declaration of competing interest

The authors declare that they have no known competing financial interests or personal relationships that could have appeared to influence the work reported in this paper.
