# Supplementary material for: Apoptotic breast cancer cells after chemotherapy induce pro-tumour extracellular vesicles via LAP-competent macrophages
Source: Redox Biol. 2024 Dec 28;80:103485. doi: 10.1016/j.redox.2024.103485 (PMC11758215; doi:10.1016/j.redox.2024.103485)
Supplement: Multimedia component 4 [file mmc4.docx]

**Supplementary Data**

**Apoptotic** **breast cancer cells after chemotherapy induce pro-tumour extracellular vesicles via LAP-competent macrophages**

Qi Zhang^1,2,3#^, Xiaodi Liu^4#^, Qiuxia Wei^1,2^, Shiyu Xiong^1,2^, Wanrong Luo^1,2^, Yingshi zhou^1,2^, Jincheng Cao^1,2^, Xiaolin Xu^1^, Rongbin Liu^1^, Xinyu Tang^5,6*^, Wenyue Zhang^1,2*^, Baoming Luo^1*^

^1^Department of ultrasound, Sun Yat-Sen Memorial Hospital, Sun Yat-Sen University, Guangzhou 510120, China.

^2^Guangdong Provincial Key Laboratory of Malignant Tumour Epigenetics and Gene Regulation, Sun Yat-Sen Memorial Hospital, Sun Yat-Sen University, Guangzhou 510120, China.

^3^Department of ultrasound, The First Affiliated Hospital with Nanjing Medical University, Nanjing, 210029, China.

^4^Department of Ultrasound, Laboratory of Ultrasound Imaging and Drug, West China Hospital, Sichuan University, Chengdu, 610041, China

^5^Department of Breast Surgery, Department of General Surgery, The First Affiliated Hospital with Nanjing Medical University, Nanjing, 210029, China.

^6^Jiangsu Key Lab of Cancer Biomarkers, Prevention and Treatment, Jiangsu Collaborative Innovation Center For Cancer Personalized Medicine, School of Public Health, Nanjing Medical University, Nanjing, 211166, China.

^#^Qi Zhang and Xiaodi Liu contributed equally to this manuscript.

^*^Corresponding author: Xinyu Tang ([nmutxy@njmu.edu.cn](mailto:nmutxy@njmu.edu.cn)); Wenyue Zhang ([zhangwy226@mail.sysu.edu.cn](mailto:zhangwy226@mail.sysu.edu.cn)); Baoming Luo ([luobm@mail.sysu.edu.cn](mailto:luobm@mail.sysu.edu.cn))

**
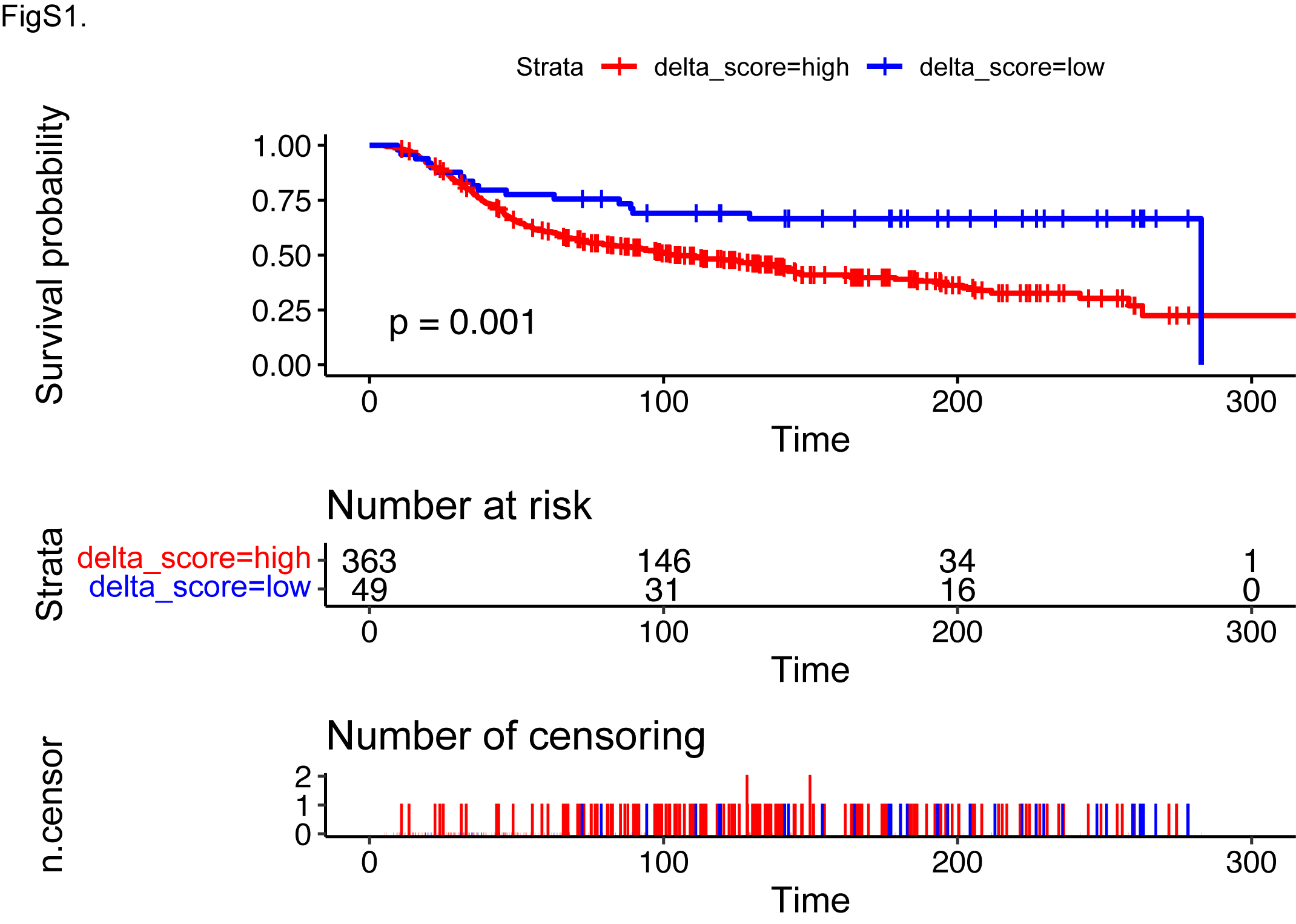
 Figure S1.** Kaplan-Meier Plot showing overall survival of breast cancer patients grouped by delta score in the Metabric cohort.

**
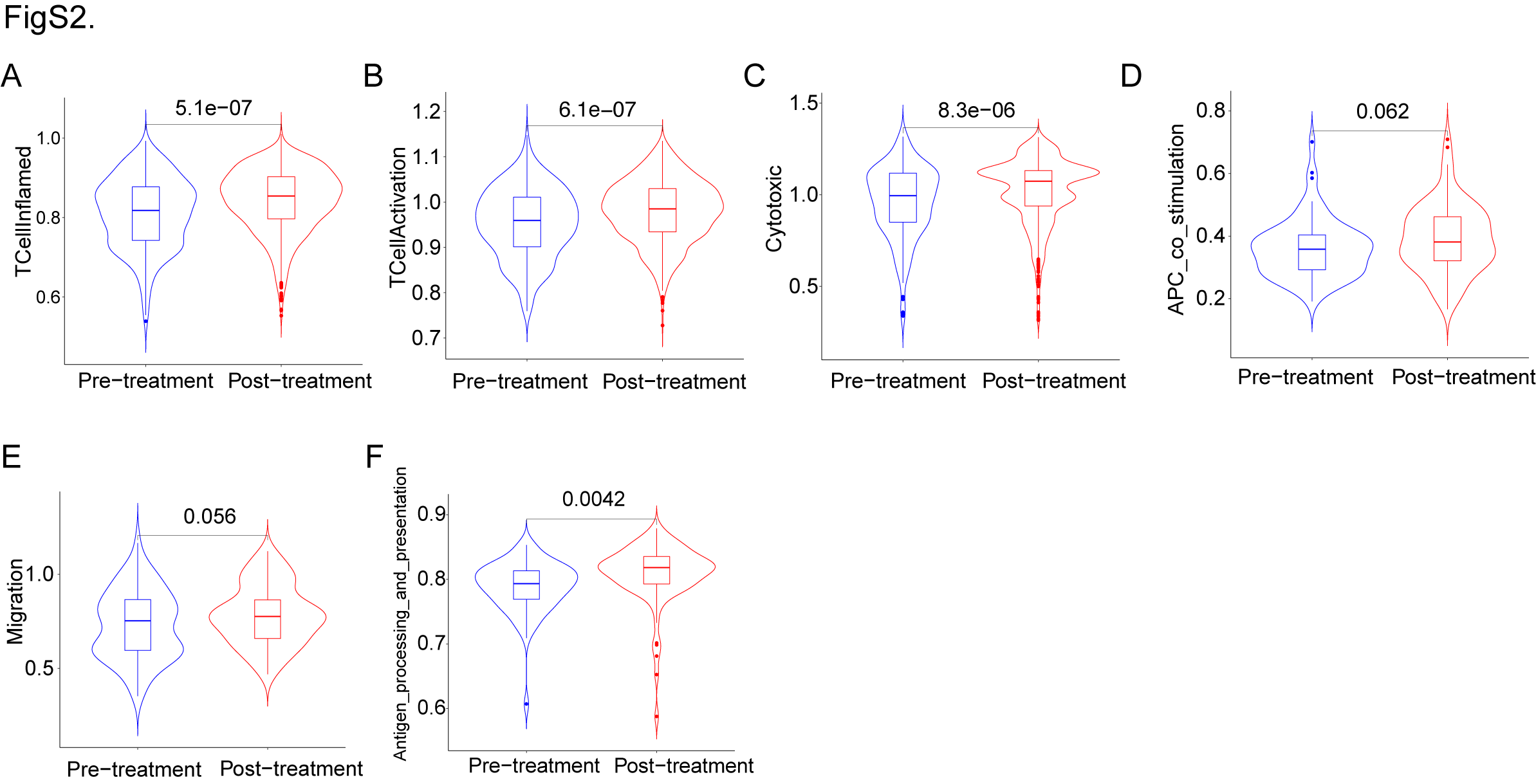
 Figure S2. A-F** Enrichment scores of T cells (A-C) and dendritic cells (D-F) before and after chemotherapy among SD patients.

**
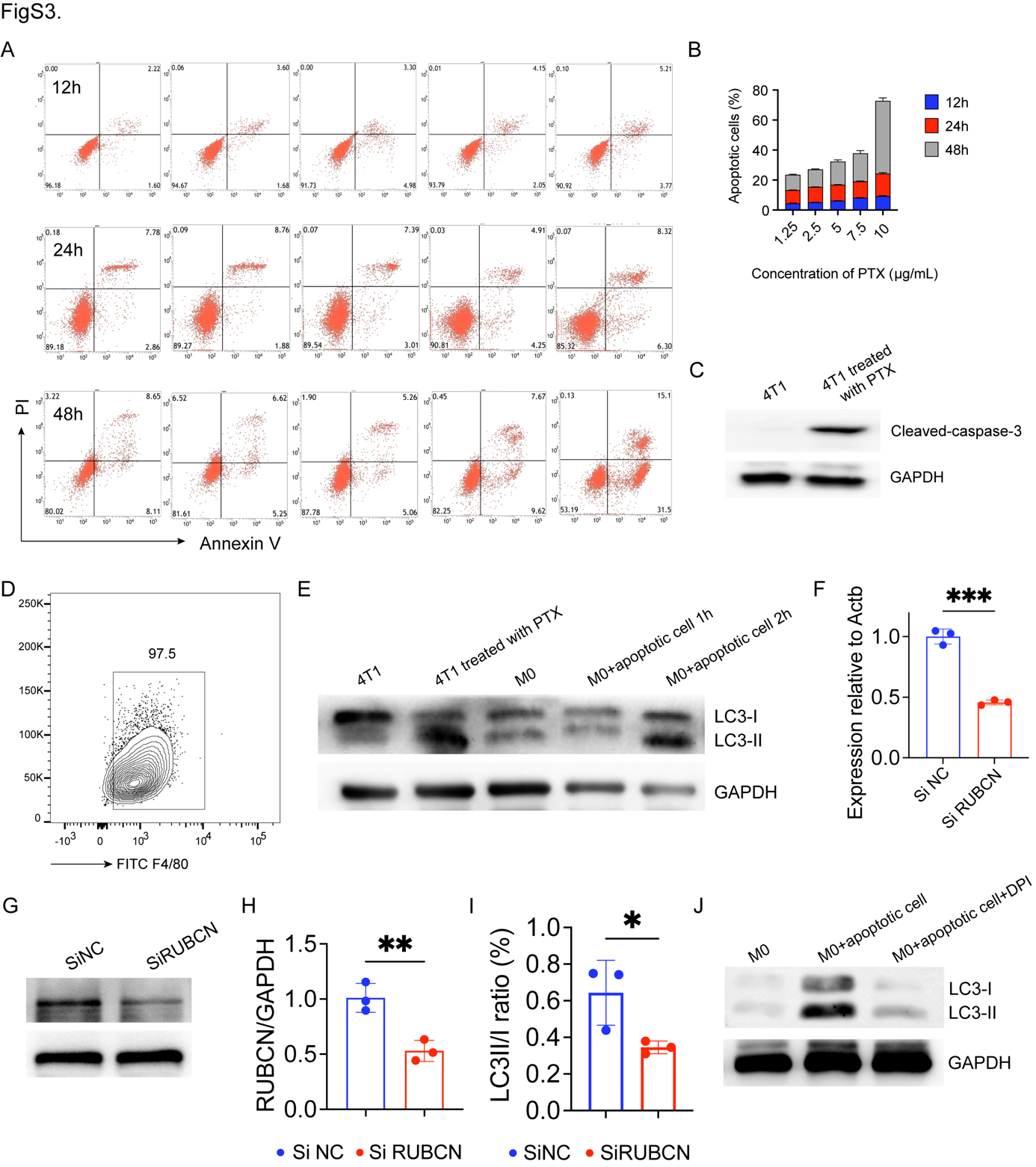
 Figure S3. A-B** Representative flow cytometric plots of BMDMs stained with Annexin V and PI (A) and bar plots of proportion of apoptotic cells in the right panel (n=3) (B). **C** Lysates from BMDMs were immunoblotted with antibody to cleaved-caspase-3. **D** Representative flow cytometric plot of BMDMs stained with anti-F4/80. **E** Lysates from BMDMs were immunoblotted with antibody to LC3B. **F** Bar plots of mRNA relative expression of RUBCN in BMDMs (n=3). **G** Lysates from BMDMs were immunoblotted with antibody to RUBCN. **H** Bar plots of grey value of RUBCN/GAPDH (n=3). **I** Bar plots of grey value of LC3II/I (n=3). **J** Lysates from BMDMs were immunoblotted with antibody to LC3B. Data are shown as mean ± SD, P > 0.05 (ns), P < 0.05 (*), P < 0.01 (**), P < 0.001 (***), P < 0.0001 (****).

**
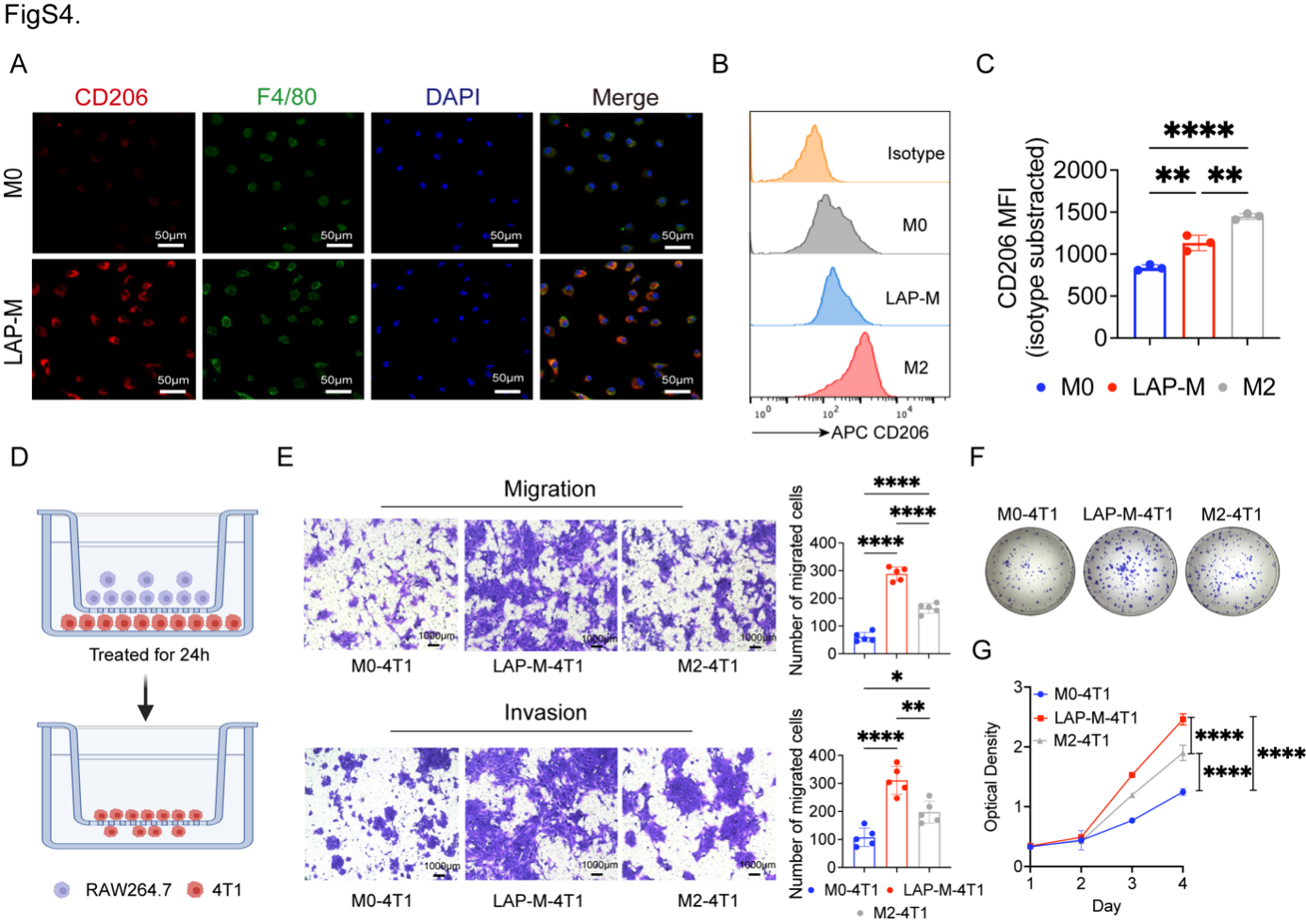
**

**Figure S4.** **A** Representative confocal pictures of CD206 (red) and F4/80 (green) in BMDMs. **B-C** Histogram plots (left panel) and bar plots (right panel) of MFI of CD206 in BMDMs (n=3). **D** Co-culture of macrophages and 4T1 cells for 24 h by a transwell system, 24 h later, tumour cells were collected for further functional assays (E-G). **E** Representative pictures of tumour invasion and migration assays with the related bar plots shown in the right panel (n=5). **F-G** Representative pictures of plate cloning assay (F) and optical density of CCK-8 assay (n=5) (G). Data are shown as mean ± SD, P > 0.05 (ns), P < 0.05 (*), P < 0.01 (**), P < 0.001 (***), P < 0.0001 (****).

**
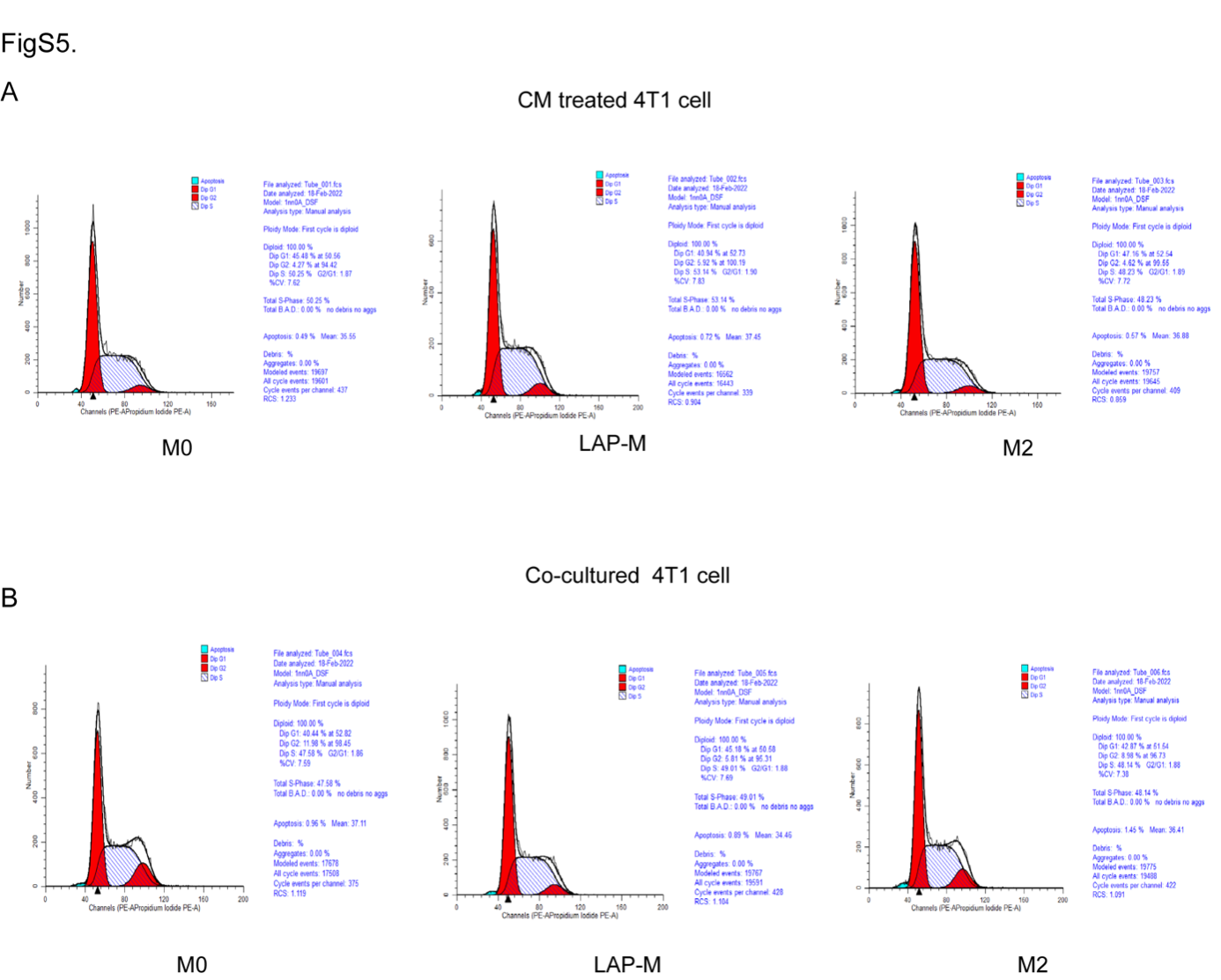
**

**Figure S5. A**-**B** Representative pictures of cell cycle for 4T1 treated with CM (A) or co-cultured with macrophages (B).

**
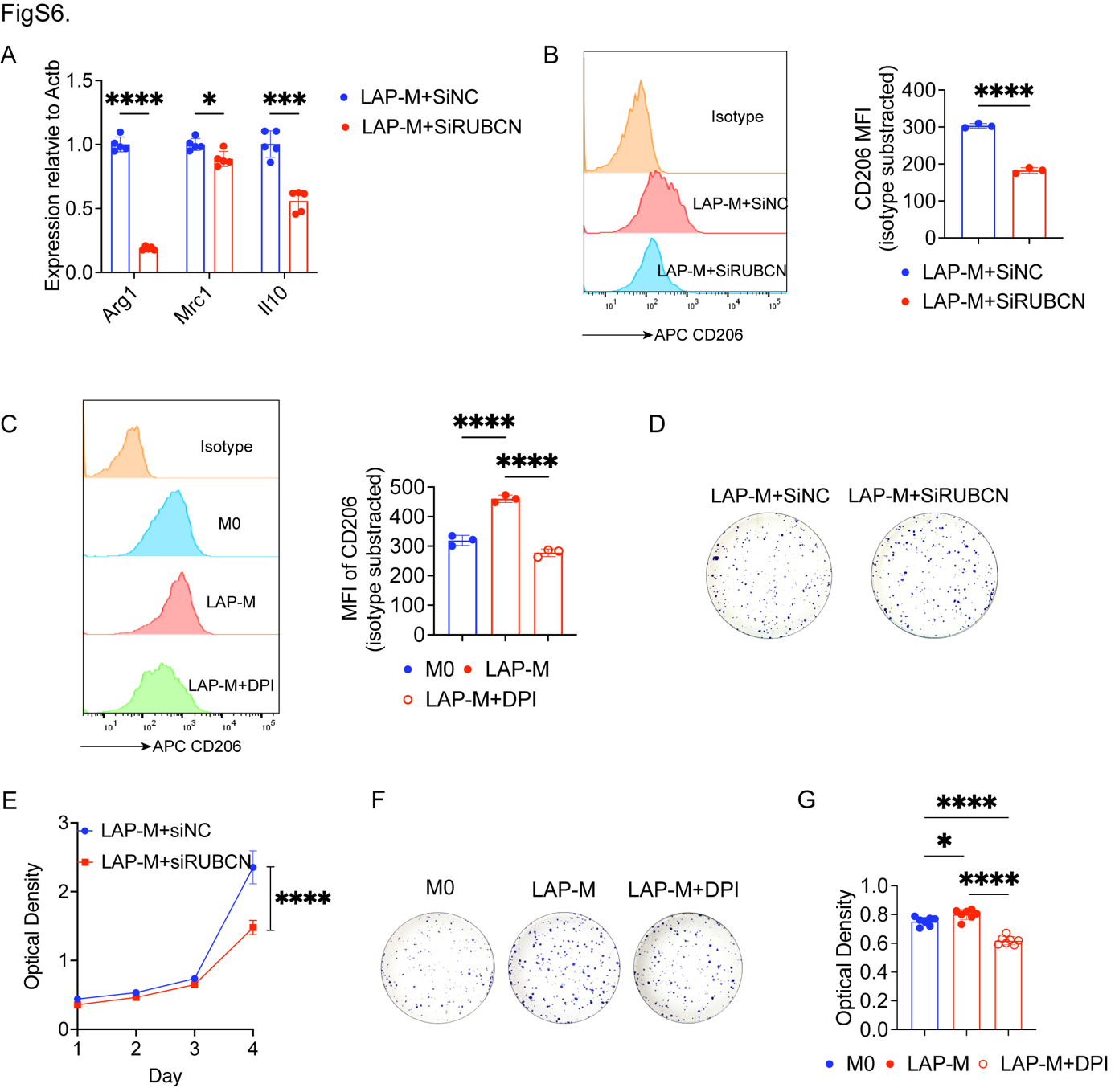
 Figure S6. A** Bar plots of mRNA relative expression of M2 genes in BMDMs (n=5). **B** Histogram plots of BMDMs stained with CD206 (left panel) and bar plots of MFI of CD206 (right panel, n=3). **C** Histogram plots of BMDMs stained with CD206 (left panel) and bar plots of MFI of CD206 (right panel, n=3). **D-E** Representative pictures of plate cloning assay (D) and optical density of CCK-8 assay (n=6) (E). **F** Representative pictures of plate cloning assay. **G** Bar plots of the third day of optical density of CCK-8 assay (n=7). Data are shown as mean ± SD, P > 0.05 (ns), P < 0.05 (*), P < 0.01 (**), P < 0.001 (***), P < 0.0001 (****).

**
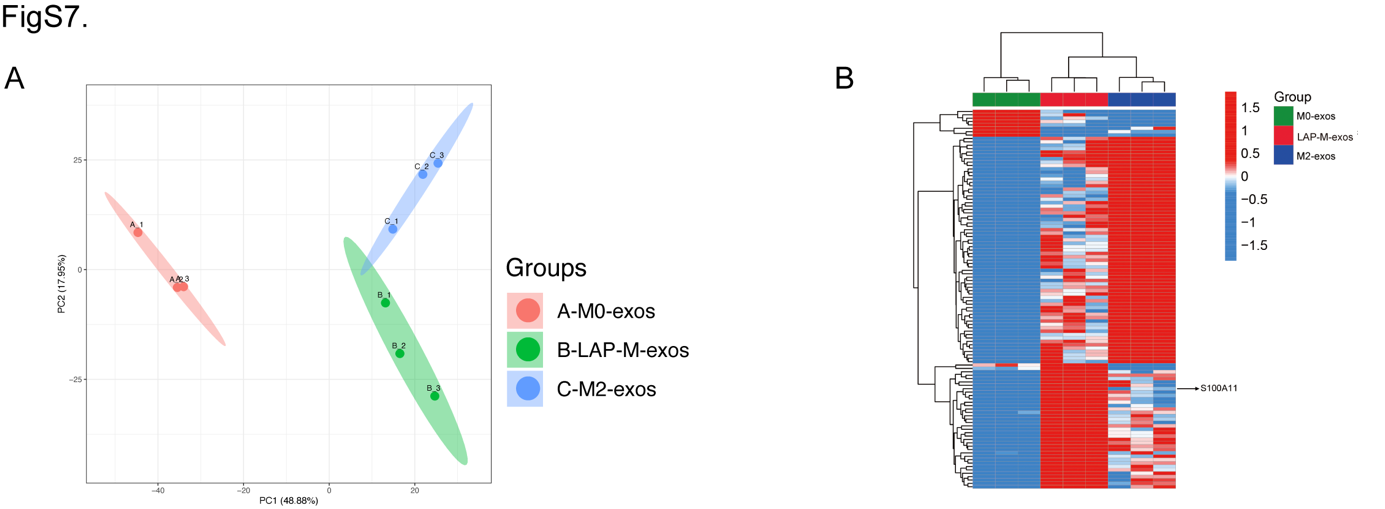
Figure S7. A** PCA analysis of exosome proteomes among the three groups. **B** Heat map represented exosomal protein expression among the three groups.

**
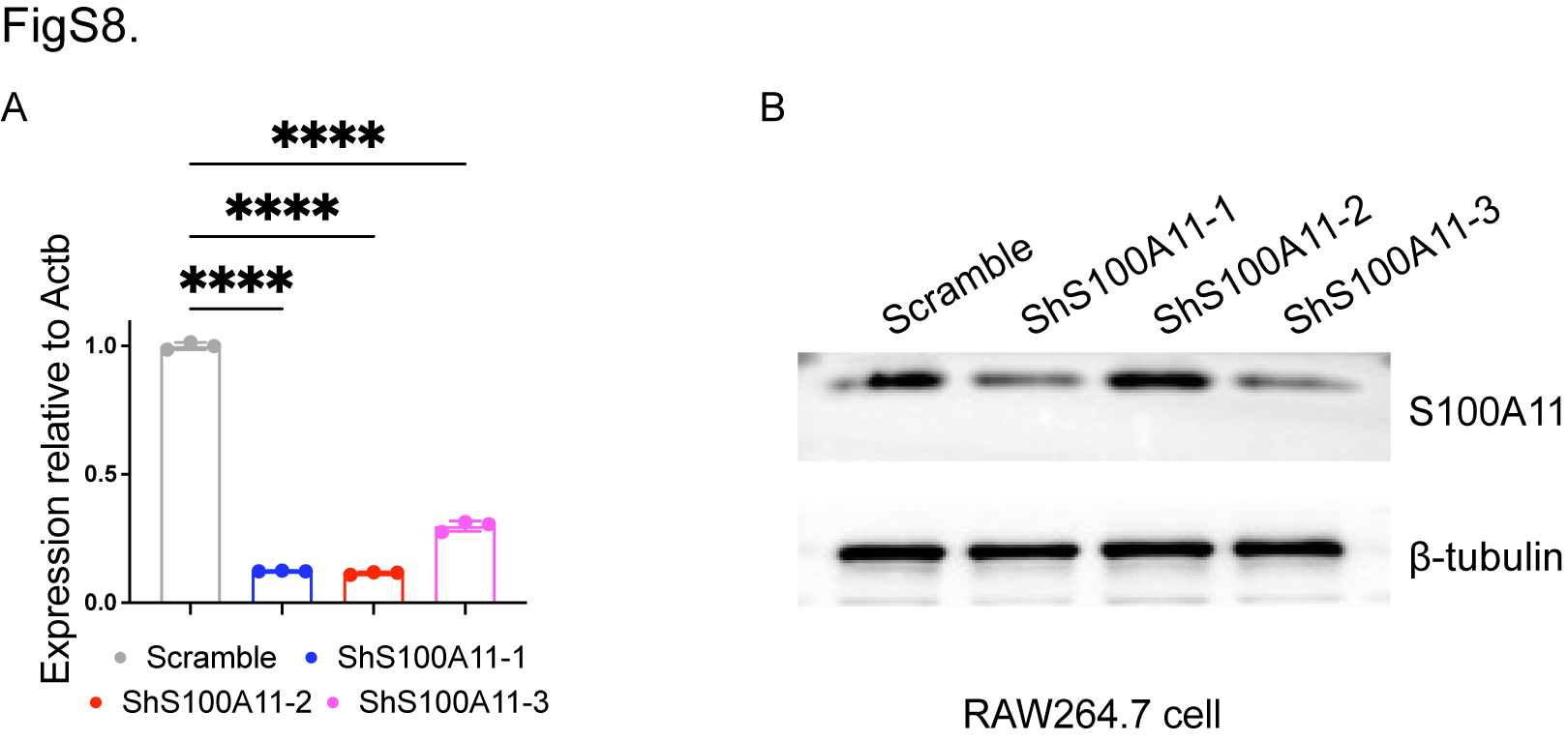
**

**Figure S8. A** Bar plots of mRNA relative expression of S100A11 in RAW264.7 cells. **B** Lysates from RAW264.7 cells were immunoblotted with antibody to S100A11.


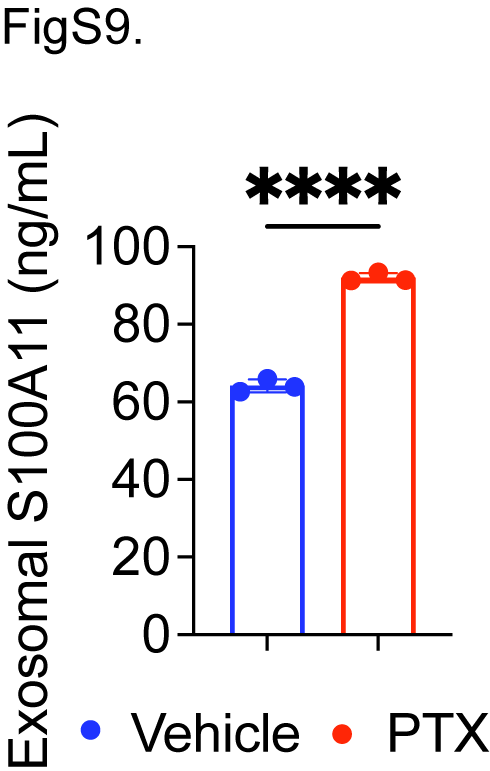


**Figure S9.** Bar plots of expression of exosomal S100A11 in peripheral blood from 4T1-bearing mice (n=3). Data are shown as mean ± SD, P > 0.05 (ns), P < 0.05 (*), P < 0.01 (**), P < 0.001 (***), P < 0.0001 (****).

**Table S1. The primer sequences of qRT-PCR**

| Gene | Sequence (5’ to 3’) Forward | Sequence (5’ to 3’) Reverse |
| --- | --- | --- |
| *Cd86* | CTGGACTCTACGACTTCACAATG | AGTTGGCGATCACTGACAGTT |
| *Mrc1* | CTCTGTTCAGCTATTGGACGC | CGGAATTTCTGGGATTCAGCTTC |
| *Rubcn* | GAAGACGACTGTGGAAGGTTTG | AGCCCATGATACAGGATGTTCT |
| *Arg1* | CTCCAAGCCAAAGTCCTTAGAG | AGGAGCTGTCATTAGGGACATC |
| *Il6* | TAGTCCTTCCTACCCCAATTTCC | TTGGTCCTTAGCCACTCCTTC |
| *Nox2* | GTTCTCAGCCCAACAATACAAGA | GTGGACGGGTCGATGTCAC |
| *Actb* | GGCTGTATTCCCCTCCATCG | CCAGTTGGTAACAATGCCATGT |
| *Il10* | GCTGGACAACATACTGCTAACC | ATTTCCGATAAGGCTTGGCAA |
| *H2-Aa* | TCAGTCGCAGACGGTGTTTAT | GGGGGCTGGAATCTCAGGT |
| *H2-Ab1* | AGCCCCATCACTGTGGAGT | GATGCCGCTCAACATCTTGC |
| *S100A11* | AAGTACAGCGGGAAGGATGGA | ATGCGGTCAAGGACACCAG |
